# Laparoscopic gastric pouch and remnant resection: a novel approach to refractory anastomotic ulcers after Roux-en-Y Gastric Bypass: Case report

**DOI:** 10.1186/1471-2482-11-33

**Published:** 2011-12-02

**Authors:** Daniel C Steinemann, Marc Schiesser, Pierre-Alain Clavien, Antonio Nocito

**Affiliations:** 1 Department of Visceral and Transplantation Surgery, University Hospital Zurich, Raemistrasse 100, 8091 Zurich, Switzerland; 2 Department of Surgery, Cantonal Hospital Bruderholz, 4104 Bruderholz, Switzerland

**Keywords:** Roux-en-Y-Gastric Bypass, bariatric surgery, anastomotic ulcer, marginal ulcer, obesity

## Abstract

**Background:**

Anastomotic or marginal ulcers occur in 0.6 to 16% of patients after laparoscopic Roux-en-Y-Gastric Bypass. Initial therapy aims at eliminating known risk factors including smoking, Helicobacter pylori infection, use of non-steroidal anti-inflammatory drugs and inhibition of gastric acid secretion. While this approach is successful in 68 to 88% of the cases, up to one third of patients need a subsequent surgical revision. However, marginal ulcers still recur in up to 10% of cases after revisional surgery, thus constituting a serious challenge for bariatric surgeons.

**Case presentation:**

We herein report a case of an insidious marginal ulcer refractory to both medical therapy with high-dosed proton pump inhibitors and sucralfate as well as surgical therapy consisting of the lengthening of a short alimentary limb and later resection of the gastroenterostomy and construction of a new tension-free anastomosis. Only after gastrectomy by laparoscopic en-bloc resection of the gastrojejunostomy, the gastric pouch and resection of the gastric remnant with reconstruction by esophagojejunostomy the patient remained free of symptoms.

**Conclusion:**

By laparoscopic resection of the entire gastric pouch and the gastric remnant the risk to leave a suboptimally vascularised or even ischemic pouch in situ was avoided. The esophagojejunostomy was then created in healthy, good vascularised tissue. In our case this novel approach was effective in the management of a refractory anastomotic ulcer and might represent a rescue option when simple revision of the gastrojejunostomy fails.

## Background

A specific complication after laparoscopic Roux-en-Y-Gastric-Bypass (LRYGB) is a marginal or anastomotic ulcer (AU) occurring at the gastrojejunal anastomosis. While AU can remain asymptomatic in 62-92% of the cases [1-4], they can frequently cause disabling pain or complications such as perforation and bleeding [5,6]. The incidence of AU varies from 0.6% to 16% in endoscopic studies [1-4].

The etiology of AU is unclear. Two classes of risk factors have been suggested: operative and patient related factors. Although large gastric pouch, vertically oriented pouch [7], gastro-gastric fistula [8], local tissue ischemia due to anastomotic tension [9] or presence of foreign bodies in the ulcer ground (e.g. nonabsorbable sutures) [10] have been previously discussed, there is still a lack of high level of evidence demonstrating these factors to be significant. In contrast, better data exist for patient related factors, showing that smoking (odds ratio (OR) 30.6), use of nonsteroidal anti-inflammatory drugs (OR 11.5) and lack of proton pump inhibitor (PPI) prophylaxis (OR 3) represent significant risk factors [11].

Therefore, ulcer therapy starts with elimination of patient related risk factors and inhibition of gastric acid secretion. While this approach is successful in 68 to 88%, up to one third of the patients need a subsequent surgical revision [8,12]. Although revision surgery is successful in most cases, AU recur in up to 8% [8], thus leading to a distressing situation for both patients and bariatric surgeons.

We herein report a case of an insidious AU refractory medical and surgical therapy, which finally required an aggressive approach consisting of laparoscopic gastric pouch and gastric remnant resection.

## Case presentation

In a 50 year male patient with a BMI of 45 kg/m^2 ^an antecolic, antegastric LRYGB with a 100 cm alimentary and a 60 cm biliary limb was performed. The gastrojejunostomy was constructed using a 25 mm circular stapler (EEA 2535, 3.5 mm Staples, Covidien^®^). Simultaneously, a 6 cm silastic (Fobi) ring was placed around the gastrojejunostomy. A few weeks after discharge, the patient, who continued smoking after surgery, presented with strong epigastric pain, postprandial regurgitation and vomiting. He was unable to eat solid food and to attend work. Endoscopy revealed two AU at the gastrojejunostomy. Oral PPI therapy (esomeprazole, 80 mg/die) was initiated and, since it was thought to be partly responsible for the symptoms, the silastic ring was removed. Intravenous high-dose PPI (esomeprazole, 240 mg/die) led to healing of the AU. However, epigastric pain and regurgitation did not ameliorate. A 99 m Tc-mebrofenin scintigraphy revealed severe biliary reflux. Seven months after LRYGB the patient was referred to our department.

At the initial consultation the patient was taking up to 600 mg/day esomeprazole and 200 mg/day tilidin orally. As an AU could not be detected further diagnostic investigations were performed:

• Upper gastrointestinal (GI) contrast series revealing a small gastric pouch without signs of a gastric fistula and normal passage.

• Double balloon push enteroscopy demonstrating a short (40 cm) alimentary limb.

• High-resolution esophageal manometry revealing a normotensive propulsive peristalsis and a normotense lower esophageal sphincter.

• 24 h-impedance pH-metry - performed under antacid medication - showing no pathological acid or non-acid reflux.

• MRI in Sellink technique showing no obstruction of the small bowel.

Apart from the biliary reflux diagnosed by scintigraphy consistent with a very short Roux-limb by push-enteroscopy, no other reason for the epigastric pain was detected. We performed a laparoscopic lengthening of the Roux limb by additional 120 cm resulting in a new alimentary limb of 160 cm. Oral PPI therapy (80 mg/day) was continued and sucralfate (4 g/d) was added.

**Figure 1:**
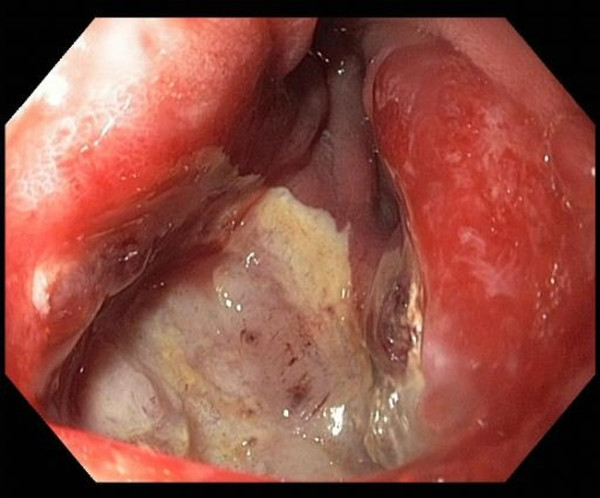
**Recurrent anastomotic ulcer in the intestinal part of the gastrojejunostomy**.

After a short period without pain and regurgitation, symptoms recurred two weeks after Roux limb lengthening. Despite PPI therapy endoscopy revealed a recurrent AU at the gastrojejunostomy (Figure 1). Therefore a laparoscopic resection of the gastrojejunostomy was performed followed by a construction of a new, tension-free anastomosis using a 25 mm circular stapler (EEA 2535, 3.5 mm Staples, Covidien^®^). PPI therapy and sucralfate were continued. Again the patient was discharged free of symptoms on postoperative day five.

One month later and one year after initial LRYGB surgery, the patient was again not free of epigastric pain. Gastroscopy showed again a large AU at the gastroenterostomy. Meanwhile, the patient was finally motivated enough to quit smoking and was enrolled in a stop-smoking program. Since gastrin level was not elevated (111 ng/l) an underlying gastrinoma could be excluded. Furthermore, Helicobacter pylori and hyperparathyroidism as additional potential causes for anastomotic ulcers were also ruled out. Nevertheless, epigastric pain and the AU persisted.

**Figure 2:**
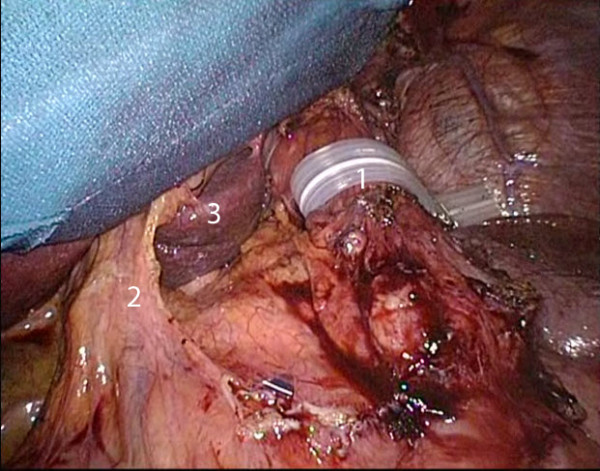
**Situs after en-bloc resection of the gastric pouch and the gastrojejunostomy**. (1 = esophagus, 2 = hepatoduodenal ligament, 3 = caudate lobe).

**Figure 3:**
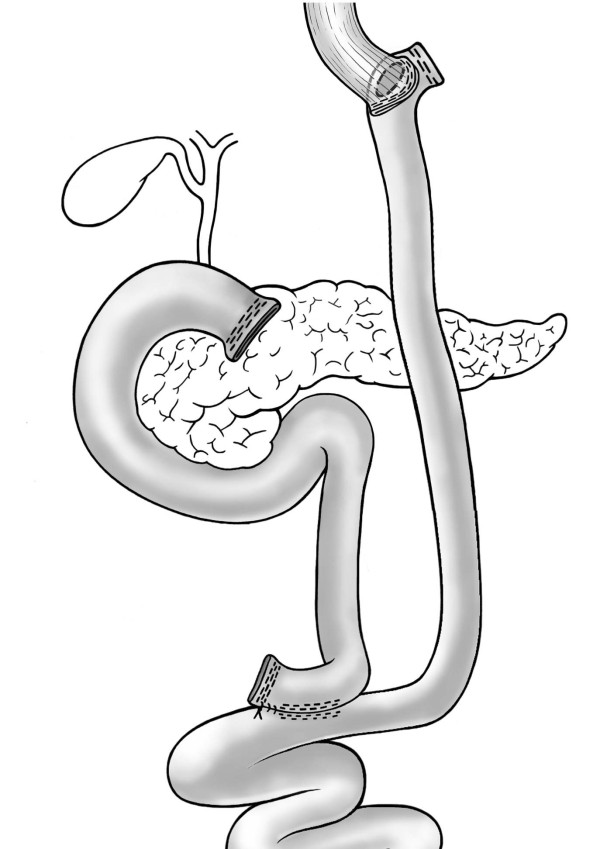
**Roux-Y reconstruction with esophagojejunostomy**.

At this point an aggressive approach was decided consisting of a gastrectomy by laparoscopic en-bloc resection of the gastrojejunostomy and the gastric pouch with transsection 2 cm proximal to the angle of His and resection of the gastric remnant (Figure 2). The gastrointestinal continuity was re-established by the construction of an esophagojejunostomy using a 25 mm circular stapler (Figure 3). Two days after surgery an upper GI contrast series showed no leakage or stenosis at the level of the esophagojejunostomy. The patient was discharged on postoperative day 10. Six months later the patient was free of symptoms, he was able to start opioid weaning and had regained 6 kg of weight. Finally, endoscopy showed a regular esophagojejunostomy.

## Discussion and conclusion

Our case of a persistently recurring AU is representative for the current shortcomings in understanding the pathogenesis and thus optimal treatment of AU. We describe a successful approach for the management of intractable AU.

After LRYGB up to 7% of patients develop upper GI symptoms. Analysis and management of this condition is often challenging as 32% of symptomatic patients show a normal anatomy at endoscopy [13]. Conservative therapy has been reported to be successful in 68%-88% of the cases [8,12]. In our case, endoscopy showed no abnormalities apart from a short Roux limb at the time of referral. As a short Roux limb may facilitate biliary reflux causing postoperative pain and AU [14], we decided to lengthen the alimentary limb. Despite improvement of regurgitation symptoms, epigastric pain and AU recurred.

Approximately one third of AU recur after medical therapy. For these cases a redo of the gastrojejunostomy with a success rate of 87% has been advised [8]. Before we embarked on this strategy, we reevaluated whether putative factors leading to a recurrence were present. Endoscopy and GI contrast series excluded potential operative risk factors. Therefore a resection of the gastrojejunostomy with subsequent PPI therapy was performed. However, the ulcer recurred potentially due to the inability of the patient to quit smoking.

After revision surgery for AU, a recurrence rate of 8% has been described. In these cases, revision of the gastrojejunostomy combined with gastric remnant resection has been advocated reducing gastrin-producing- and parietal cells [8].

In contrast to the proposed simple revision of the anastomosis, we opted for a laparoscopic resection of the entire gastric pouch and the gastric remnant. By this means, the risk to leave a suboptimally vascularised or even ischemic pouch in situ was avoided since the resection was taken back to esophageal tissue. The circular esophagojejunostomy was then created in healthy, good vascularised tissue. Subsequently, the patient was free of symptoms and no recurrence was observed after a follow-up of 6 months. Hence, in our case this novel approach was effective in the management of a refractory AU and might represent a rescue option when simple revision of the gastrojejunostomy fails.

## Competing interests

The authors declare that they have no competing interests. No financial support has been received.

## Consent

Written informed consent was obtained from the patient for publication of this case report and any accompanying images. A copy of the written consent is available for review by the Editor-in-Chief of this journal.

## Authors' contributions

DCS drafted and finalized the manuscript, MS and PAC reviewed the manuscript, AN performed the surgery, monitored the drafting and critically reviewed the manuscript and has given final approve for publication. All authors read and approved the final manuscript.

## Pre-publication history

The pre-publication history for this paper can be accessed here:

http://www.biomedcentral.com/1471-2482/11/33/prepub
